# From simulated empathy to structural attunement: Realtime Editable Memory Topology and the evolution of emotionally grounded AI

**DOI:** 10.3389/frai.2026.1749517

**Published:** 2026-03-31

**Authors:** John Albanese

**Affiliations:** 1 Independent Researcher, Bristol, CT, United States; 2 ESPN Inc, Bristol, CT, United States

**Keywords:** affective computing, autobiographical memory, conversational AI, graph-based memory architectures, memory-augmented language models, structural adaptation

## Abstract

Large language models (LLMs) and retrieval-augmented generation (RAG) systems have achieved remarkable linguistic fluency, and many now implement persistent cross-session memory at the application layer. However, these mechanisms typically rely on external storage and reinjection of stored content rather than structural reorganization of memory relationships. As a result, they remain limited in their ability to integrate affective salience into a dynamically evolving internal memory topology capable of supporting coherent long-term behavior. To address this gap, we introduce Realtime Editable Memory Topology (REMT), an architectural framework for imbuing conversational agents with persistent autobiographical memory organized as an evolving graph of emotionally valenced nodes. REMT formalizes synthetic neuroplasticity through explicit update rules governing edge reinforcement, decay, and pruning, and introduces a bounded Mood Index that modulates retrieval bias and response generation as a function of accumulated affective experience. In this Perspective, we argue that memory-grounded architectures integrating insights from cognitive science, affective computing, and memory-augmented neural systems are necessary for building adaptive conversational agents with stable long-term interactional tendencies. We conclude by outlining a roadmap for empirical validation using an internally developed evaluation framework, with results to be reported in a future Original Research article.

## 1 Introduction

Large language models (LLMs) have achieved remarkable fluency in open-ended dialogue, yet they remain limited in their capacity for structural emotional continuity. While commercial systems such as ChatGPT, Claude, and Gemini now implement persistent cross-session memory, these mechanisms typically rely on external memory injection—storing user facts or interaction logs without performing intrinsic structural reorganization of memory relationships. Such approaches enable contextual recall but generally treat memory as a repository of stored entries rather than a dynamically evolving topology shaped by affective experience.

Attempts to extend continuity have also relied on retrieval-augmented generation (RAG) (Johnson et al., 2017; Pinecone Systems, 2023) or expanded context windows (Alselwi et al., 2025). These methods enable systems to resurface information, but they primarily operate through retrieval and context extension rather than topology-level adaptation. They can recall facts but do not typically restructure memory associations as a function of emotional salience or narrative reinforcement. Even advanced persistent systems therefore remain limited in their capacity for affect-driven structural adaptation.

This gap points to a deeper architectural need: an AI system must be able to encode and reorganize experience in ways that mirror autobiographical memory. In humans, memory is not a static archive; it is a dynamic structure shaped by emotional salience, reinforcement, and decay. We argue that grounding artificial systems in analogous structural principles is essential for creating agents capable of coherent long-term behavior in relational settings.

To explore this possibility, we introduce *Realtime Editable Memory Topology (REMT)*, a framework designed to construct persistent, emotionally weighted autobiographical memory within artificial systems. Whereas, existing persistent-memory implementations provide cross-session storage and retrieval mechanisms, REMT models experience as an evolving graph in which connections are continuously reweighted through synthetic neuroplasticity. The novelty of this architecture lies in its closed feedback loop between affective salience and graph restructuring: rather than merely accumulating stored entries, REMT performs topology-level adaptation through dynamic edge updates. From this structure arise properties such as continuity across sessions, mood-modulated retrieval, and individualized behavioral adaptation—outcomes driven by structural memory dynamics rather than explicit scripting or passive logging.

This Perspective situates REMT within emerging work on memory-augmented agents, including Generative Agents (Park et al., 2023); Reflexion (Shinn et al., 2023), episodic-transformer memory architectures (Fountas et al., 2024), and affect-aware conversational systems (Zhang et al., 2024). Rather than presenting experimental results, we focus on the architectural shifts required to move AI from passive persistence mechanisms toward systems capable of affect-driven structural adaptation and long-term coherence.

## 2 Background and motivation

Conversational AI has evolved rapidly from rule-based systems to large-scale generative models capable of open-domain dialogue and contextual reasoning. Contemporary LLM-based systems now implement persistent cross-session memory, storing user facts and preferences across interactions. These deployments demonstrate that modern systems incorporate cross-session storage mechanisms at the application layer. However, in most implementations, long-term memory operates through external storage and reinjection of retrieved content into the prompt context. Continuity is achieved through additive records rather than structural reorganization of memory relationships.

Efforts to extend contextual persistence have traditionally centered on retrieval-augmented generation (RAG) pipelines (Johnson et al., 2017; Pinecone Systems, 2023) and long-context transformers (Alselwi et al., 2025). These approaches improve factual recall and task consistency by resurfacing previously stored information. More recent agent-oriented systems, such as MemGPT (Packer et al., 2023), introduce explicit memory management mechanisms including paging and compartmentalization strategies for long-term interaction histories. While effective for state tracking and retrieval efficiency, these systems treat memory as a collection of entries that are accessed, summarized, or replaced. The relational structure between memories is not itself dynamically reorganized as a function of affective salience.

Earlier work in memory-augmented neural architectures—including Memory Networks (Weston et al., 2015) and Neural Turing Machines (Graves et al., 2014)—demonstrated how differentiable external memory modules could extend neural computation. These architectures established important precedents for learnable read-write memory. Classical symbolic cognitive architectures such as ACT-R (Anderson et al., 1998) and Soar (Laird, 2012) similarly modeled structured memory and procedural reasoning within rule-based systems. However, neither symbolic nor neural memory-augmented frameworks integrated affective salience into a continuously reorganizing memory topology coupled to generative neural language models. Their primary objectives centered on reasoning performance and task execution rather than modeling autobiographical continuity or affect-driven structural adaptation across extended human-AI interaction.

Parallel research in affective computing has emphasized the role of emotion in shaping cognition and memory. Foundational contributions by Picard (1997) established emotion-aware computation as a field, while appraisal-based models such as the OCC framework (Ortony et al., 1988) formalized how emotional evaluation influences memory organization and behavioral tendencies. In human cognition, emotionally salient experiences are preferentially reinforced, reorganized, and integrated into evolving autobiographical structures. Recent work on affect-aware language agents (Zhang et al., 2024) reflects growing interest in continuous affect modeling, yet most implementations modulate response tone rather than structurally reorganizing internal memory representations.

A related and rapidly developing body of research examines personality expression and identity consistency in large language models. Recent studies have evaluated persona fidelity using psychometric and situational judgment frameworks (Yost et al., 2025) and explored deterministic personality expression and structured personality adaptation mechanisms in LLM agents (Kruijssen and Emmons, 2025; Wang et al., 2026). These approaches address behavioral consistency and trait alignment at the interaction level, often through explicit trait conditioning or diagnostic control structures. However, they do not typically formalize how affective salience reshapes the structural topology of long-term memory itself.

Taken together, these strands of research highlight a remaining architectural gap. Existing persistent memory systems excel at storage, retrieval, and state tracking. Memory-augmented neural architectures and classical cognitive architectures formalize structured memory representations. Affective computing provides formal models of emotional appraisal. Personality-oriented LLM research explores trait stability and adaptive persona expression. What remains underspecified is an integrated mechanism in which affective salience directly reshapes the structural topology of long-term memory and conditions future retrieval dynamics.

Realtime Editable Memory Topology (REMT) is motivated by this gap. Rather than coupling a language model to a static graph database augmented with sentiment scores and time-based decay, REMT formalizes memory as a closed, self-modifying system in which affective salience governs structural reweighting of inter-node relationships. The novelty lies not in the presence of individual components—semantic similarity, valence scoring, or exponential decay—but in their formal integration into a unified update rule that continuously reshapes the topology of memory itself. In REMT, retrieval bias, mood modulation, and edge reinforcement are mathematically coupled, such that changes in affective activation alter future structural connectivity, which in turn conditions subsequent retrieval and response generation. Continuity therefore emerges from topology-level adaptation rather than additive logging or external memory injection.

## 3 Core architecture of REMT

The preceding limitations suggest that persistence and emotional grounding cannot be appended as auxiliary features; they must be embedded within the memory architecture itself. Realtime Editable Memory Topology (REMT) addresses this requirement by modeling memory as a continuously evolving graph of symbolic and affective experience. Each interaction contributes to a structured autobiographical topology that reorganizes dynamically over time.

### 3.1 Memory representation

Each user-agent interaction is encoded as a memory node containing:

- A semantic embedding vector capturing linguistic content.
- A normalized emotional valence score *E*_*i*_∈[−1, 1].
- Symbolic tags representing abstract thematic abstractions.
- Temporal metadata.

Edges between nodes represent temporal proximity, emotional similarity, and symbolic association. The resulting graph integrates informational and affective structure into a unified autobiographical representation.

### 3.2 Structural initialization

When a new node *v*_*i*_ is inserted, its initial edge weight relative to node *v*_*j*_ is defined in Equation 1 as:


$$\begin{array}{l} w_{ij}\left(t_{0}\right)=\lambda_{s}\text{sim}\left(v_{i},v_{j}\right)\\ \;+\lambda_{e}\text{aff}\left(v_{i},v_{j}\right)\\ \;+\lambda_{n}\text{narr}\left(v_{i},v_{j}\right)\\ \;-\lambda_{d}\Delta t_{ij} \end{array}$$ (1)

Here, sim(*v*_*i*_, *v*_*j*_) denotes cosine similarity between embedding vectors in the reference implementation. Alternative similarity metrics (e.g., dot-product similarity or learned metric functions) are conceptually compatible but are not used in the present formulation. aff(*v*_*i*_, *v*_*j*_) denotes affective proximity, narr(*v*_*i*_, *v*_*j*_) captures symbolic overlap, and Δ*t*_*ij*_ represents normalized temporal distance.

The coefficients λ_*k*_ are bounded hyperparameters such that λ_*k*_∈[0, 1] and $\underset{k}{\sum }\lambda_{k}=1$. In the reference implementation, these weights are selected via constrained grid search on held-out interaction sequences to balance semantic similarity and affective proximity while preserving graph stability (i.e., preventing unbounded edge growth or premature pruning). Alternative adaptive or learned weighting schemes may be explored in future implementations.

### 3.3 Synthetic neuroplasticity

After initialization, edge weights evolve over discrete timesteps.

When a node participates in retrieval or response generation, its associated edges are reinforced as shown in Equation 2:


$$\begin{array}{l} w_{ij}\left(t+1\right)=w_{ij}\left(t\right)+\eta \text{ salience}\left(v_{j}\right) \end{array}$$ (2)

When inactive, edges decay exponentially according to Equation 3:


$$\begin{array}{l} w_{ij}\left(t+1\right)=w_{ij}\left(t\right)e^{-\alpha } \end{array}$$ (3)

Edges falling below threshold ϵ for sustained duration τ are pruned.

This separation of reinforcement and decay improves clarity while preserving formal equivalence to a piecewise update rule.

### 3.4 Mood Index

The system's global affective state is represented by a scalar Mood Index in Equation 4:


$$\begin{array}{l} M_{t}=\frac{1}{N}\overset{N}{\underset{i=1}{\sum }}E_{i}A_{i}S_{i} \end{array}$$ (4)

where:

- *E*_*i*_∈[−1, 1] is emotional valence,
- *A*_*i*_∈[0, 1] is activation level,
- *S*_*i*_∈[0, 1] is symbolic reinforcement weight,
- *N* is the number of active emotional nodes.

Under these bounded definitions, *M*_*t*_∈[−1, 1].

Affective proximity is defined in Equation 5 as:


$$\begin{array}{l} \text{aff}\left(v_{i},v_{j}\right)=1-|E_{i}-E_{j}| \end{array}$$ (5)

Node salience used in reinforcement updates is defined in Equation 6 as:


$$\begin{array}{l} \text{salience}\left(v_{j}\right)=|E_{j}|A_{j}S_{j} \end{array}$$ (6)

**Figure 1:**
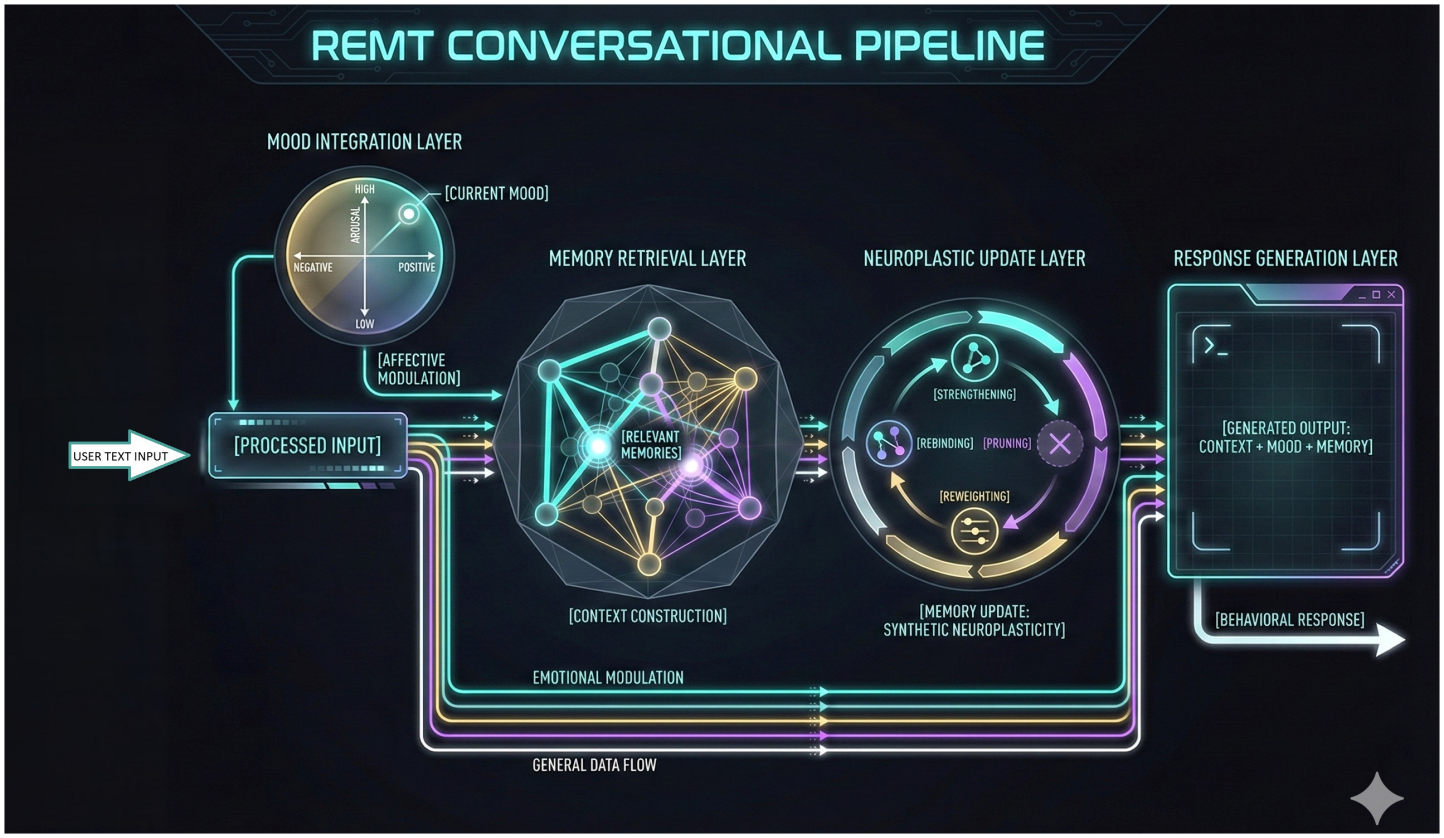
REMT conversational pipeline. Diagram illustrating the sequential REMT processing flow from user text input through mood integration, memory retrieval, neuroplastic update, and response generation. Arrows indicate data flow and modulation across the pipeline, including affective weighting, relevant memory retrieval, synthetic neuroplasticity, and the generation of behaviorally conditioned output.

The Mood Index modulates retrieval bias by weighting affective similarity during query construction, enabling stable behavioral modulation across extended interaction sequences. The overall REMT conversational pipeline is illustrated in Figure 1.

### 3.5 Scalability

As interaction histories expand, the REMT topology grows linearly with encoded exchanges. Tractability is maintained through:

- Edge pruning to prevent unbounded growth,
- Activation-bounded retrieval over a restricted subgraph,
- Localized updates yielding *O*(*k*) complexity with respect to activated nodes.

These constraints preserve computational feasibility while supporting long-term structural adaptation.

## 4 Emergent behavior and the Mood Index

### 4.1 Continuity of interactional state

As the REMT topology expands through sustained interaction, distinctive behavioral patterns are expected to emerge—patterns of response consistency, affective bias, and relational style that are not explicitly scripted but arise from the structure of the memory graph itself. Because each exchange is written into an evolving autobiographical topology, REMT-based systems are designed to maintain continuity across sessions rather than resetting conversational context. Preferences, metaphors, and affective framing may recur with regularity, producing stable interactional tendencies over time.

Recent research has examined persona fidelity, psychometric evaluation, and structured personality control in large language models (Yost et al., 2025; Kruijssen and Emmons, 2025; Wang et al., 2026). These approaches demonstrate that trait-consistent behavior can be induced or measured at the interaction level. REMT differs in emphasis by focusing not on externally specified trait profiles, but on how affectively weighted autobiographical structure itself shapes continuity over time.

**Figure 2:**
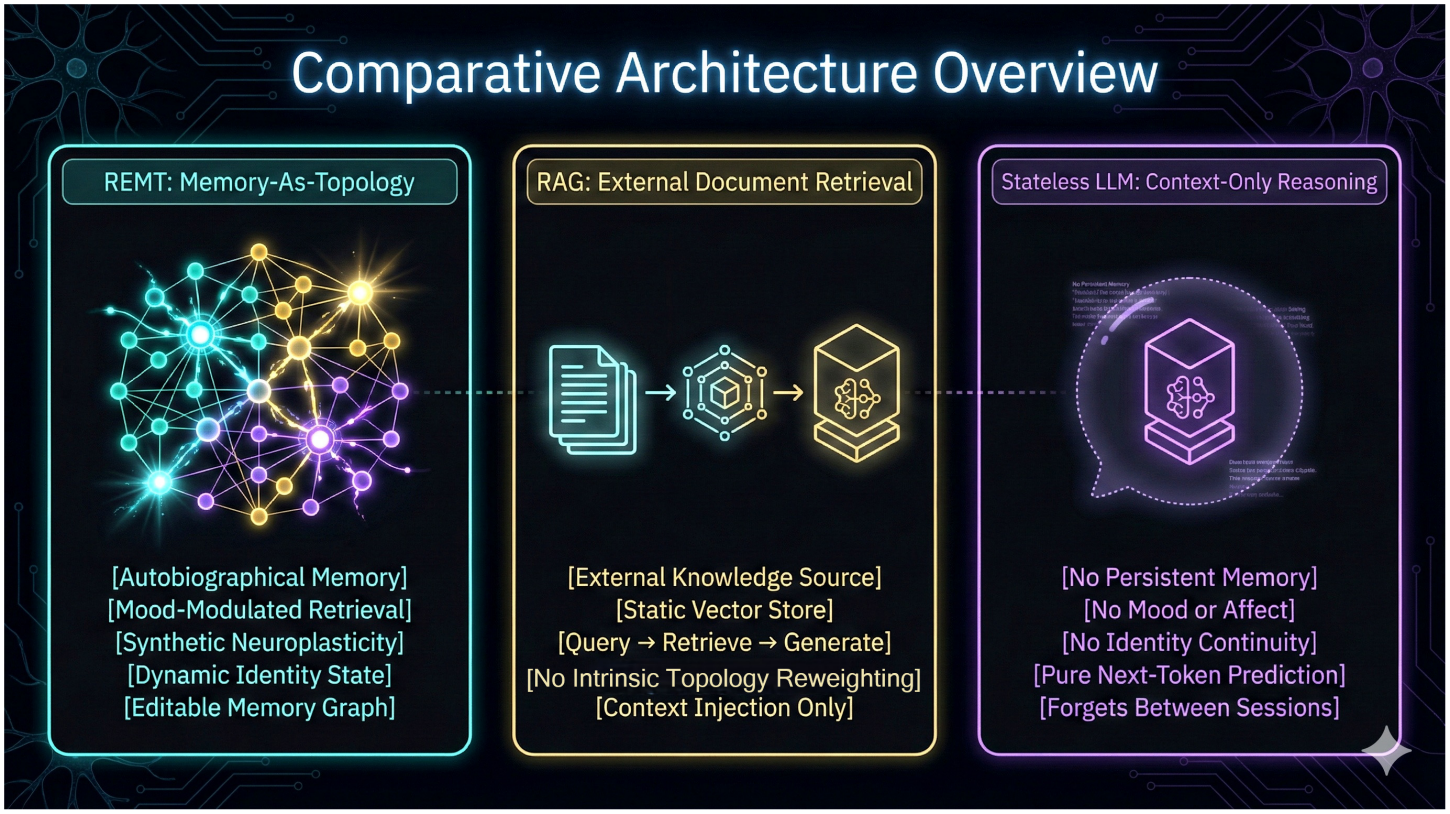
Comparative architecture overview. Diagram comparing REMT, retrieval-augmented generation (RAG), and stateless LLM architectures. REMT is presented as a memory-as-topology framework with autobiographical memory, mood-modulated retrieval, synthetic neuroplasticity, dynamic identity state, and an editable memory graph. RAG is shown as relying on external knowledge sources and static vector stores for query-time retrieval, while stateless LLMs are shown as context-only systems without persistent memory, mood, or identity continuity.

For example, if prior interactions consistently associate a user's creative activity with positive valence, subsequent retrieval may preferentially surface related framing without requiring hard-coded rules. In this architecture, continuity arises because emotionally and symbolically salient clusters exert persistent influence on future retrieval, forming an implicit attractor within the memory space that evolves gradually rather than discontinuously. A comparative overview of REMT, RAG, and stateless LLM architectures is shown in Figure 2.

### 4.2 Affective trajectory modeling

Through synthetic neuroplasticity, REMT enables the system to model affective trajectories across interaction histories. Recurrent emotional sequences—such as frustration followed by resolution or anxiety preceding achievement—can become structurally encoded within the topology, biasing future retrieval and response selection. This capacity allows the system to condition responses not only on instantaneous input but on inferred patterns across time. The resulting behavior resembles empathic adjustment, not through explicit emotional simulation, but through emotionally weighted prediction grounded in prior interactional structure. Tone, pacing, and response framing are modulated by the Mood Index, which reflects the aggregated valence of recently activated memory nodes.

### 4.3 Individualized structural differentiation

Over extended interaction, REMT-based memory topologies are expected to diverge across users. Differences in symbolic associations, affective valence distributions, and reinforcement patterns lead to individualized memory structures even when systems are initialized with identical parameters. As a result, retrieval biases and response tendencies gradually specialize in ways that reflect each interaction history. This process supports personalization without requiring per-user rule sets or manually tuned profiles, as differentiation arises from the cumulative effects of emotional weighting and structural plasticity within the memory graph.

### 4.4 Dynamic affective regulation

The Mood Index functions as a global regulatory variable linking internal memory state to external response characteristics. Variations in the index influence lexical breadth, initiative, and response restraint, providing a mechanism for smooth modulation rather than abrupt emotional shifts. Because each interaction updates the Mood Index, system behavior is governed by a closed feedback loop in which recent experience continuously informs subsequent output. This design contrasts with scripted affective overlays or static trait conditioning by maintaining internal coherence across interaction sequences while remaining responsive to contextual change.

### 4.5 Synthesis

Taken together, these mechanisms illustrate how REMT's architectural components—emotional weighting, symbolic clustering, and mood-modulated retrieval—can give rise to consistent interactional tendencies without explicit personality scripting. Rather than prescribing fixed traits or relying solely on external persona constraints, the system adapts its behavior through accumulated experience, producing continuity grounded in structural memory dynamics. These properties suggest a pathway toward conversational agents capable of long-term coherence while remaining responsive, adaptable, and aligned with evolving relational context.

## 5 Implications and future directions

### 5.1 From simulated empathy to structural attunement

REMT reframes emotional intelligence not as an aesthetic enhancement but as an architectural property of memory organization. Contemporary conversational systems can generate contextually appropriate responses and may implement persistent cross-session storage at the application layer. However, these mechanisms do not typically restructure internal memory relationships as a function of affective salience. In contrast, architectures with affectively weighted, topologically adaptive memory are designed to condition behavior on prior emotional context and consequence. By encoding affective dimensions of experience into a mutable memory topology, REMT proposes a mechanism for emotional continuity grounded in structural adaptation rather than surface-level simulation. This shift—from simulated affect to structural attunement—has implications for domains in which trust, consistency, and long-term context influence outcomes, including education, coaching, and mental health support.

### 5.2 Continuity as both capability and risk

Persistent memory can contribute to behavioral stability by reducing contradictory responses and reinforcing awareness of prior interactional context. A system that conditions output on accumulated affective and narrative history is less likely to repeat locally harmful patterns in isolation. However, this same persistence introduces meaningful risks that must be addressed at the architectural level. A memory system capable of modeling emotional trajectories could, if misused, be exploited for emotional manipulation, persuasion, or coercion. Likewise, long-term affective profiling may foster unhealthy dependency or over-attribution of agency if boundaries between user and system are insufficiently clear.

Emotional continuity should therefore be understood not as an inherent safety guarantee but as a dual-use capability whose effects depend on governance, transparency, and constraint. REMT highlights the need for safeguards that limit cross-context misuse of affective memory, enforce scope boundaries on retrieval, and prevent emotional optimization objectives from overriding user autonomy or wellbeing.

### 5.3 Ethical and societal considerations

As AI systems acquire persistent autobiographical structure, ethical responsibilities expand beyond conventional data protection. Affective records —capturing emotional valence, symbolic associations, and inferred trajectories—are more sensitive than factual interaction logs. Their storage and use raise questions of informed consent, transparency, and the right to revision or deletion. Users must be able to understand when emotional data are retained, how they influence behavior, and under what conditions they are accessed or modified.

Architectural transparency and user control mechanisms are therefore essential complements to emotional continuity. Designers must also ensure that affective realism does not blur human-machine boundaries or encourage relational dependence. Addressing these concerns requires explicit design constraints rather than *post hoc* ethical framing, reinforcing the importance of integrating safety considerations into memory architecture itself.

### 5.4 Applications and collaborative potential

When appropriately constrained, emotional continuity enables new forms of human-AI collaboration. In education, REMT-based tutors could adapt instruction based on a learner's motivational trajectory rather than static performance metrics. In healthcare and mental health support, relational agents could maintain contextual continuity across sessions while operating within defined ethical and privacy boundaries. In creative domains, systems with affective memory may help preserve narrative tone or aesthetic intent over extended projects. Across these applications, REMT shifts conversational AI from isolated task execution toward interaction histories that support sustained engagement.

### 5.5 Future empirical validation

The REMT framework is currently undergoing exploratory evaluation using MLE-Star, a proprietary machine learning evaluation environment independently developed and operated by the author on Google Cloud infrastructure. MLE-Star is not a publicly distributed Google product but a controlled experimental environment designed for systematic behavioral analysis. The framework is used to probe behavioral stability, representational structure, and longitudinal response dynamics under standardized testing conditions.

Evaluation focuses on three proposed metrics: (1) identity drift, assessing the stability of response tendencies across extended interaction sequences; (2) graph modularity, measuring the emergence and persistence of symbolic-emotional clusters within the evolving topology; and (3) mood correlation, quantifying alignment between inferred user affect and response modulation. These measures situate REMT within broader research on memory-augmented conversational agents (Park et al., 2023; Fountas et al., 2024; Zhang et al., 2024).

Results from these evaluations will be reported in a future Original Research article. The aim of this exploratory phase is to characterize the behavioral and computational properties of structurally persistent memory architectures while maintaining a clear distinction between functional continuity and phenomenological interpretation.

## 6 Conclusion

Large language models have achieved remarkable advances in linguistic prediction. Although contemporary deployments increasingly support cross-session persistence at the application layer, behavior remains largely shaped by prompt-level context unless supported by structured mechanisms for long-term memory reorganization. As a result, coherence across extended interaction histories depends not only on stored information but on how memory relationships are structured and updated over time. Realtime Editable Memory Topology (REMT) addresses this limitation by introducing an architectural framework in which experience is encoded, reorganized, and retrieved through an affectively weighted memory topology. By integrating symbolic structure, affective signals, and synthetic neuroplasticity, REMT enables conversational systems to condition behavior on accumulated interaction history rather than isolated turns.

This Perspective has argued that continuity in conversational AI need not rely solely on expanded context windows or passive retrieval mechanisms. Instead, structural memory architectures—capable of reinforcement, decay, clustering, and mood-modulated retrieval—provide a pathway toward long-term behavioral consistency without reliance on hard-coded personality models. REMT illustrates how such mechanisms can support stable interactional tendencies while remaining adaptive to new information and evolving relational context.

Beyond immediate applications, REMT contributes to ongoing discussions about the role of memory in artificial intelligence. Rather than treating intelligence as a purely reactive mapping from input to output, the framework emphasizes experience-dependent structural organization as a core architectural principle. Ongoing empirical evaluation will focus on characterizing the behavioral and computational properties of this approach, including stability, scalability, and safety. Together, these efforts aim to clarify how persistent, topology-level memory architectures can support coherent long-term interaction while remaining transparent, controllable, and aligned with human values.

## Data Availability

The original contributions presented in the study are included in the article/supplementary material, further inquiries can be directed to the corresponding author.
